# Supplementary material for: The Solute Carrier MFSD1 Decreases the Activation Status of β1 Integrin and Thus Tumor Metastasis
Source: Front Oncol. 2022 Feb 8;12:777634. doi: 10.3389/fonc.2022.777634 (PMC8861502; doi:10.3389/fonc.2022.777634)
Supplement: Supplementary file 1 [file DataSheet_1.docx]

Supplementary Material


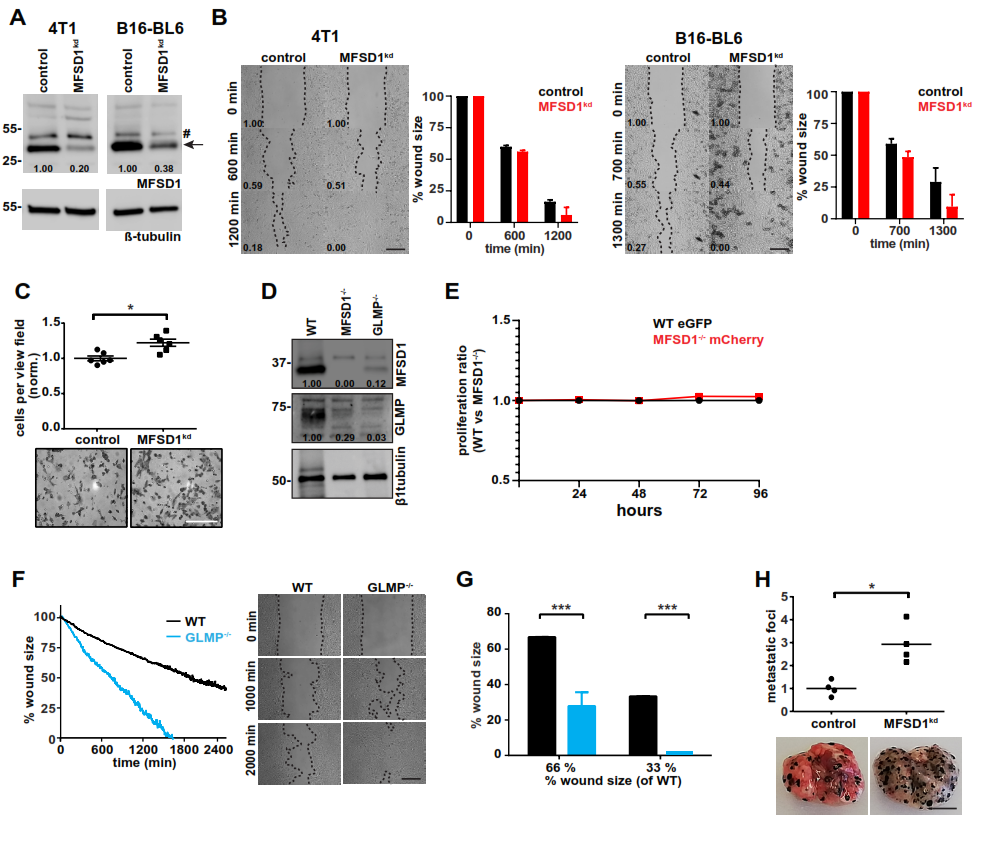


**Supplementary Figure 1. MFSD1 suppresses cell migration.**

**A)** Western blot of 4T1 and B16-BL6 cells expressing non-target shRNA (control) or MFSD1 shRNA (MFSD1^kd^). Arrow indicates MFSD1 band, # indicates unspecific band. The numbers at the bottom of the blot represent the densitometric analysis of the MFSD1 band.

**B)** Wound closing assay of 4T1 (left panel) and B16-BL6 (right panel) control and MFSD1^kd^ cells. Data from one representative experiment are shown and the analysis of each cell type is shown next to the images (n=2). The relative size of the wound remaining is indicated at the bottom left of each individual image. Bar = 0.2 mm.

**C)** Matrigel invasion assay of MC-38 control and MFSD1^kd^ cells, with one respective view field, is shown (n=6).

**D)** Western blot of MC-38 WT, MFSD1^-/-^, and GLMP^-/-^ cells. The numbers at the bottom of the blots represent the densitometric analysis of the MFSD1 and GLMP band, respectively.

**E)** Proliferation index of MC-38 WT and MFSD1^-/-^ cells analyzed by flow cytometry. In this particular experiment, WT cells expressed eGFP and MFSD1^-/-^ cells expressed mCherry (n=3). Similar results were obtained with WT mCherry and MFSD1^-/-^ eGFP cells (data not shown).

**F)** Wound closing assay of MC-38 WT and GLMP^-/-^ cells. Graph depicting the continuous shrinking of the wound (left panel) with pictures at defined time-points (right panel). Data from one representative experiment are shown. Bar = 0.2 mm.

**G)** Analysis of wound closing assay of MC-38 WT and GLMP^-/-^ cells (n=3). The % wound size left of GLMP^-/-^ cells when WT cells have moved to shrink the wound to either 66% or 33% of the original size is depicted.

**H)** Experimental metastasis with B16-BL6 control and MFSD1^kd^ cells. The relative number (normalized to the mean seen in the control) of macroscopic metastatic foci per lung is shown (n=4). Bar = 0.5 cm. * = p<0.05; *** = p<0.001.


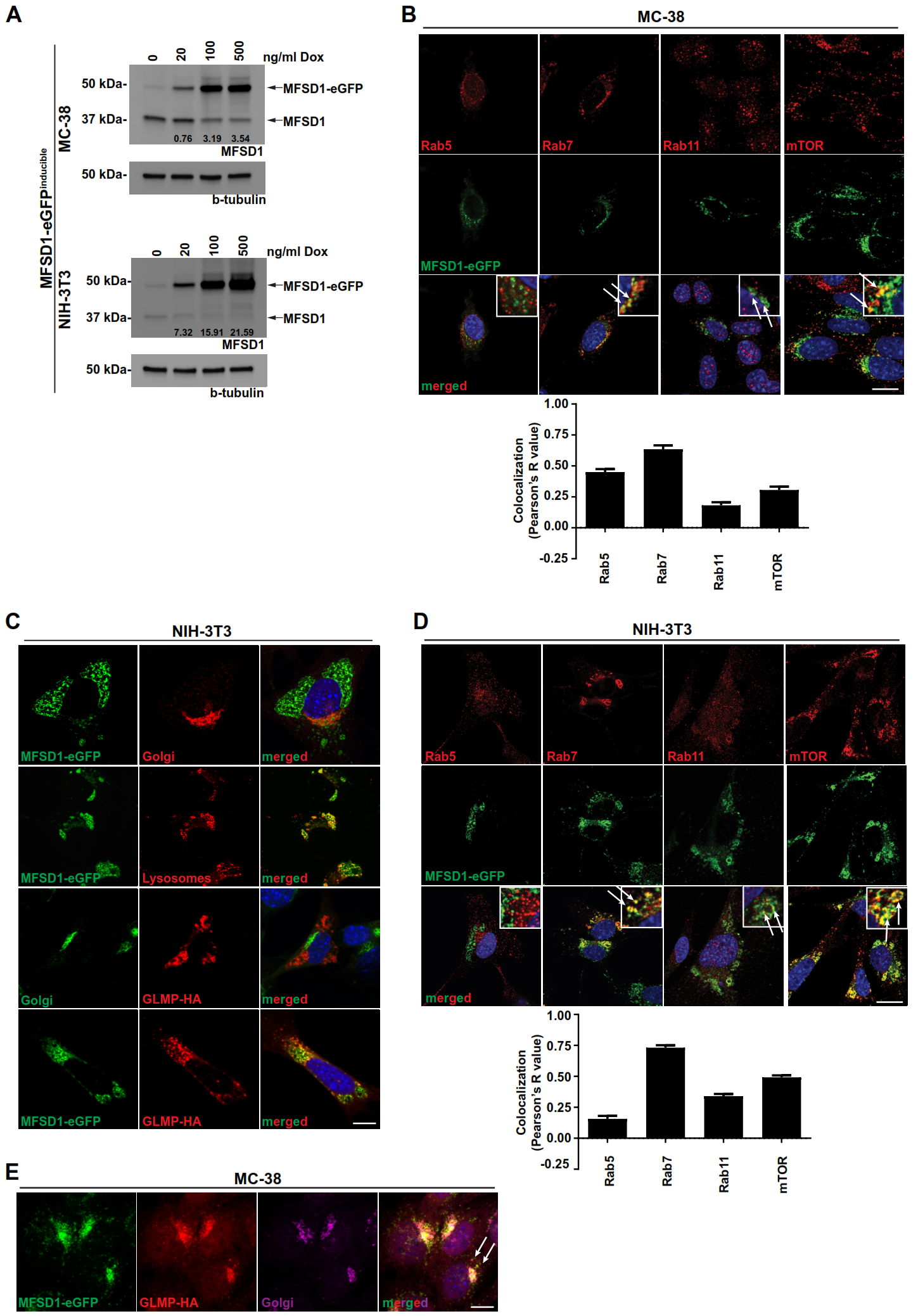


**Supplementary Figure 2. MFSD1-eGFP localizes to late-endosomes and lysosomes in MC-38 tumor cells and NIH-3T3 fibroblasts.**

**A)** MFSD1-eGFP expression was induced by different Doxycycline concentrations for 24 hours in MC-38 and NIH-3T3 cells, and the expression of MFSD1-eGFP was analyzed by Western blotting. The numbers at the bottom of the blots represent the ratio of the densitometric analysis of the MFSD1-eGFP divided by that for the endogenous MFSD1 band.

**B)** Immunofluorescence pictures of MC-38 MFSD1-eGFP cells with indicated markers, including colocalization analysis. Arrows indicate co-localization with indicated markers.

**C, D)** Immunofluorescence pictures of NIH-3T3 MFSD1-eGFP cells with indicated markers, including colocalization analysis. The Golgi compartment was stained with an antibody against giantin and lysosomes were stained with LysoTracker Red DND-99. Arrows indicate co-localization with indicated markers.

**E)** Immunofluorescence pictures of MC-38 MFSD1-eGFP and GLMP-HA cells with Golgi-apparatus marker. Arrows indicate vesicular co-localization of MFSD1-eGFP and GLMP-HA. Nuclei were counter stained with DAPI and are highlighted in blue. Bar = 10 µm. Arrows indicate co-localization.


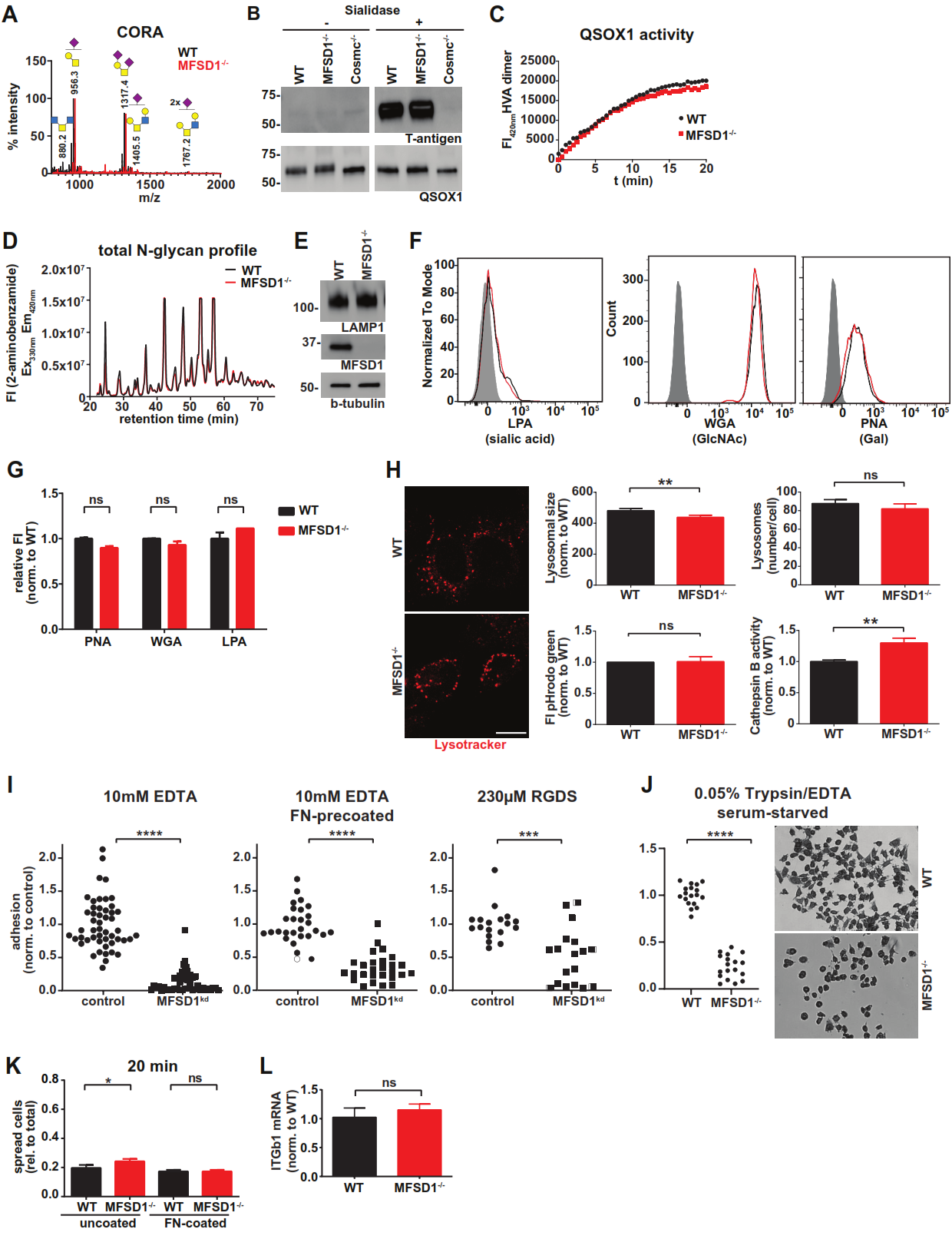


**Supplementary Figure 3. MFSD1 does not strongly affect glycosylation nor lysosomes, but rather the adhesive strength of MC-38 tumor cells.**

**A)** CORA (Cellular O-Glycome Reporter/Amplification) analysis by mass spectrometry of secreted O-glycans by MC-38 WT and MFSD1^-/-^ cells. The MFSD1^-/-^ diagram was shifted to the right to visualize the identical relative abundance of sialyl- and disialyl- T antigen in WT and MFSD1^-/-^ MC-38 cells.

**B)** Western blot of purified QSOX1-StrepTagII purified from supernatant of MC-38 WT, MFSD1^-/-^, and Cosmc^-/-^ cells (n=2).

**C)** QSOX1 activity measurement. The result of one out of two experiments is shown.

**D)** LC profile of total N-glycans isolated from MC-38 WT and MFSD1^-/-^ cells.

**E)** Western blot of LAMP1 in MC-38 WT and MFSD1^-/-^ cells.

**F-G)** FACS analysis of cell surface lectin staining in MC-38 WT and MFSD1^-/-^ cells (n=2 for LPA; n=3 for PNA and WGA).

**H)** LysoTracker staining of MC-38 WT and MFSD1^-/-^ cells. The listed parameters were analyzed by ImageJ. Lysosomal size (n>200), lysosomes number/cell (n>50), pHrodo green (n=4), Cathepsin B activity (n=6). Bar = 10 µm.

**I-J)** MC-38 cell detachment with the indicated chemicals. Per experiment 3 view fields per replicate (three replicates per experiment) were analyzed. Adhesion was determined by the area covered by still adherent cells present on the cell culture plates, within experiments adhesion was normalized to that of the WT cells.

**K)** Spreading assay of MC-38 WT and MFSD1^-/-^ on uncoated and FN-coated plates at 20 min time point (n≥21 view fields).

**L)** qPCR analysis of β1-integrin transcription in MC-38 WT and MFSD1^-/-^ cells. GAPDH served as a control (n=3). * = p<0.05; ** = p<0.01; *** = p<0.001; **** = p<0.0001; ns = not significant.


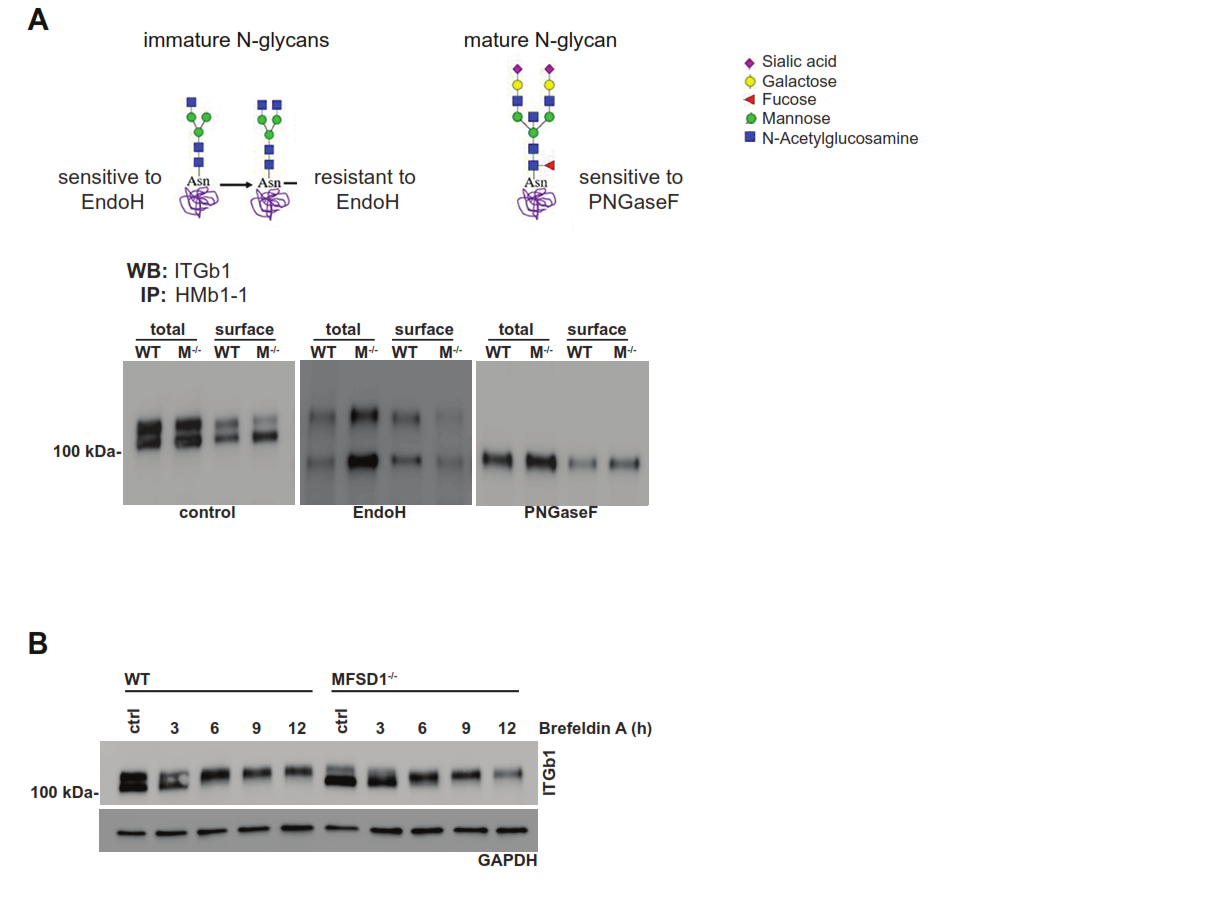


**Supplementary Figure 4. Immaturely N-glycosylated β1-integrin is sensitive to EndoH treatment.**

**A)** Schematic of N-glycans senstitive or resistant to EndoH or PNGaseF glycosidases (top panel). Western blot of immunoprecipitated β1-integrin from cell lysates (total) or the cell surface of MC-38 WT and MFSD1^-/-^ cells treated with glycosidase EndoH, PNGaseF, or untreated (control) (bottom panel) (n=1).

**B)** Western blot of Brefeldin A treated MC-38 WT and MFSD1^-/-^ cell lysates (n=2).


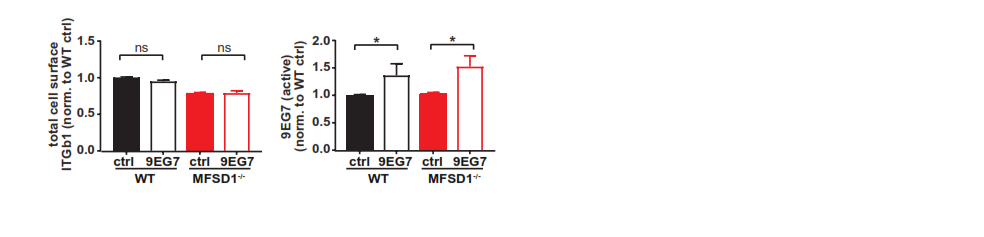


**Supplementary Figure 5. The increased β1-integrin activation index enables pro-metastatic phenotypes.**

**A)** Analysis of flow cytometry stainings of MC-38 WT and MFSD1^-/-^ cells treated with active conformation-specific β1 integrin antibody clone 9EG7 (n=4). Left panel shown total cell surface β1 integrin; right panel shows β1 integrin of active conformation on the cell surface. These data underlie Figure 5C.


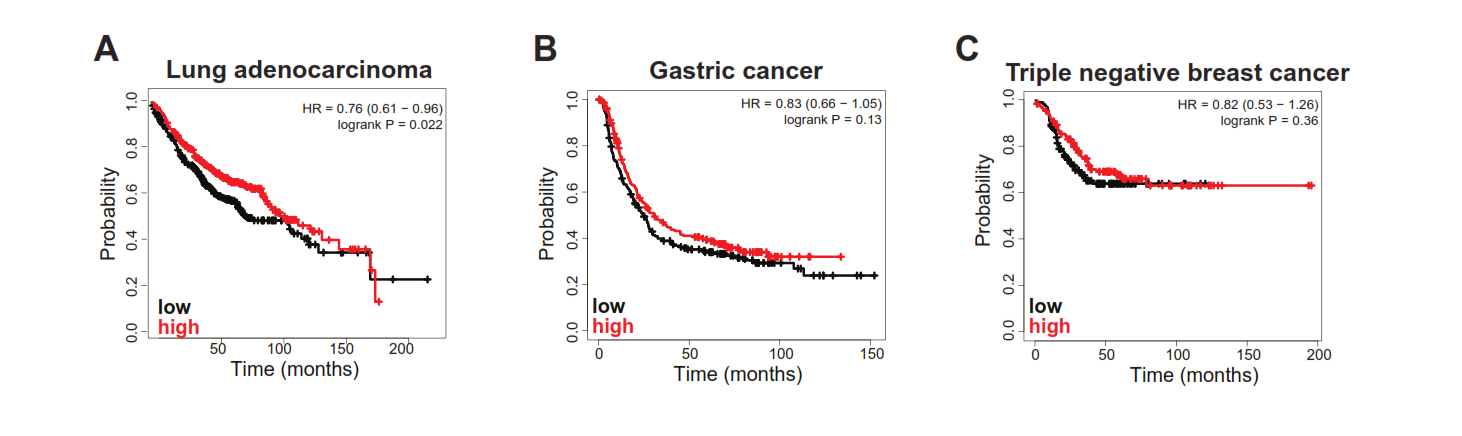


**Supplementary Figure 6. Reduced β1-integrin expression levels tend to correlate with poor patient prognosis.**

**A-C)** KM Plotter graph of respective patient prognosis. Lowest (black line) or highest quartile (red line) expression levels of β1-integrin are shown.
